# Association of *Bifidobacterium*-regulated NIS expression with the CD40-NF-κB pathway in Graves’ disease

**DOI:** 10.3389/fmicb.2026.1894196

**Published:** 2026-08-19

**Authors:** Kexin Shi, Xinxin Wan, Zhe Ying, Zhengwei Wen, Xiang Li, Huanbin Li

**Affiliations:** 1 Department of Nuclear Medicine, The First Affiliated Hospital of Wenzhou Medical University, Wenzhou, Zhejiang, China; 2 Wenzhou Key Laboratory of Sanitary Microbiology, Key Laboratory of Laboratory Medicine, Ministry of Education, School of Laboratory Medicine and Life Sciences, Institute of One Health, Wenzhou Medical University, Wenzhou, Zhejiang, China; 3 Wenzhou Medical University Renji College, Wenzhou Medical University, Wenzhou, Zhejiang, China

**Keywords:** *Bifidobacterium*, CD40, Graves’ disease, NF-κB, NIS

## Abstract

**Introduction:**

Graves’ disease (GD) represents a typical autoimmune thyroid disease triggered by thyroid-stimulating autoantibodies. Accumulating studies have verified the close correlation between intestinal microecology and GD progression, with *Bifidobacterium* prominently implicated, yet the specific molecular mechanisms underlying this association remain elusive. This study aimed to explore the regulatory effects of *Bifidobacterium adolescentis* on GD development via the CD40-NF-κB signaling cascade and sodium/iodide symporter (NIS) modulation.

**Methods:**

Clinically, fecal specimens from GD patients were recruited before and after iodine-131 (^131^I) therapy to characterize dynamic changes in intestinal *Bifidobacterium* abundance. *In vitro* cellular assays were conducted on human thyroid cells stimulated with *Bifidobacterium adolescentis*, followed by detection of cell proliferative activity, CD40-NF-κB pathway activity and NIS protein expression. *In vivo* GD mouse models were administered with *Bifidobacterium adolescentis* intervention, and subsequent assessments included thyroid and colonic histopathological changes and gut microbial profiling. The expression levels of key proteins associated with the CD40-NF-κB pathway and NIS were also quantified in thyroid and colon tissues.

**Results:**

Clinical results demonstrated that *Bifidobacterium adolescentis* abundance was significantly higher in GD patients prior to ^131^I treatment and markedly decreased after therapy. *In vitro*, *Bifidobacterium adolescentis* treatment facilitated thyroid cell proliferation, elevated NIS expression and activated the CD40-NF-κB signaling in a dose-dependent manner. In animal models, *Bifidobacterium adolescentis* supplementation aggravated hyperthyroidism, thyroid hyperplasia and intestinal inflammatory injuries.

**Discussion:**

Mechanistically, *Bifidobacterium adolescentis* drove CD40 and NIS upregulation through triggering NF-κB pathway activation. Collectively, *Bifidobacterium adolescentis* exacerbates GD pathogenesis by activating the CD40-NF-κB signaling axis and modulating thyroid NIS expression. This work reveals a novel microbial regulatory mechanism of GD, offering a promising theoretical basis for developing gut microbiota-targeted therapeutic strategies for GD treatment.

## 1 Introduction

Graves’ disease (GD), a prototypical organ-specific autoimmune disorder, is characterized by antibody-mediated pathogenesis arising from complex interactions between genetic susceptibility and environmental triggers (Chaker et al., 2024; Davies et al., 2020; Yang and Chen, 2025). Fundamental to thyroid hormone synthesis, the sodium-iodide symporter (NIS) mediates the critical first step of iodide uptake (Eleftheriadou et al., 2020). Current therapies have limitations: antithyroid drug requires prolonged treatment with high relapse rates; surgery carries inherent risks like recurrent laryngeal nerve injury; Iodine-131 (^131^I) therapy could causes permanent hypothyroidism (Stan and Dosiou, 2025; Uludag et al., 2024; Zhao et al., 2024). These therapeutic challenges emphasize the critical need for the development of precision-targeted therapies that address underlying pathogenic mechanisms while minimizing treatment-related morbidity.

Gut dysbiosis, a common phenomenon in individuals with GD, is characterized by significant alterations in the richness, diversity, and composition of intestinal bacteria (Yang et al., 2024; Fang et al., 2024). These changes are closely associated with the disease stage, thyroid autoantibody levels, and treatment response (Liu et al., 2024; Jiang W. et al., 2021). Interestingly, *Bifidobacterium* has demonstrated a notable influence on GD. Intriguingly, certain research suggests that *Bifidobacterium* may promote hyperthyroidism (Zufry et al., 2024; Gong et al., 2021; Yang et al., 2022). However, there is currently no direct evidence establishing a causal relationship between GD and *Bifidobacterium*.

A genome-wide association study found that the CD40 gene is associated with the onset and progression of GD (Jiang H. et al., 2021). CD40, is expressed in immune cells as well as in some non-immune cells, including thyroid cells. Aberrant signaling through the CD40 has been implicated in the pathogenesis of several autoimmune diseases (Lu et al., 2022; Wagner et al., 2023). Recent studies have shown that CD40 may act via the NF-κB pathway, which regulates thyroid-specific genes like NIS (Lee et al., 2017; Reale et al., 2016; Reale et al., 2018; Nicola et al., 2010). Furthermore, *Bifidobacterium* has recently been shown to regulate iodide-binding capacity through its metabolites (Liao et al., 2025). We hypothesize GD patients exhibit significant gut microbiota changes before and after ^131^I treatment, and *Bifidobacterium* may regulate NIS expression through the CD40-NF-κB pathway.

## 2 Materials and methods

### 2.1 Patients and data

Consecutive patients diagnosed with Graves’ disease (GD) who received ^131^I radiotherapy at the First Affiliated Hospital of Wenzhou Medical University were enrolled in this study. The study protocol was approved by the Ethics Committee of the First Affiliated Hospital of Wenzhou Medical University (approval number: KY2025-R30). Written informed consent was obtained from all participants or their parents.

Inclusion criteria were defined as follows: (1) elevated serum triiodothyronine (T3) and thyroxine (T4) levels with reduced thyroid-stimulating hormone (TSH) concentration; (2) positive thyroid-stimulating hormone receptor antibody (TRAb); (3) diffuse thyroid enlargement.

Exclusion criteria included: previous radioiodine treatment; a history of gastrointestinal diseases; recent administration of antibiotics, steroids, immunosuppressants, prebiotics, or probiotics.

All participants were requested to provide fecal samples 1–2 days before ^131^I treatment and one month after treatment. Samples were collected in the morning following an overnight fast (≥8 h) and transported to the laboratory within 2 h using cryogenic ice. 16S rRNA gene sequencing was performed to identify and classify bacterial species.

### 2.2 Thyroid cell and experimental design

#### 2.2.1 Cell culture and bacterial culture

The human normal thyroid cell line Nthy-ori3-1 was purchased from Shanghai Jinyuan Biotechnology Co. Nthy-ori3-1 cells were routinely cultured in RPMI-1640 medium (Sigma-Aldrich, R6504, China) supplemented with 10% fetal bovine serum (FBS, Invitrogen, Waltham, MA, USA) and 1% penicillin–streptomycin (100 U/mL penicillin and 100 μg/mL streptomycin, Beyotime, China). Cells were maintained in an incubator with 5% CO2 at 37 °C. *Bifidobacterium* adolescentis strains were isolated from Live *Bifidobacterium* Capsules. The strains were anaerobically cultured in De Man, Rogosa, and Sharpe (MRS) broth (Oxoid, CM11153B, China) supplemented with 0.05% L-cysteine (Serva, 17769, China) at 37 °C under anaerobic conditions using an AnaeroPack (Mitsubishi Gas Chemical Company, Japan) with a gas mixture of 95% N₂ and 5% H_2_. Prior to experiments, bacterial concentration was determined using a spectrophotometric microplate reader (BioTek, Winooski, Vermont, USA) at OD₆₀₀, with absorbance values correlated to colony-forming unit (CFU) counts on MRS agar plates following 48 h of anaerobic incubation. Bacteria were then washed with phosphate-buffered saline (PBS, pH 7.4, Sigma-Aldrich, P4474, China) by centrifugation (3,000 × g, 10 min). A bacterial suspension containing 10^8^CFU in 50 μL PBS was prepared for subsequent experiments. Nthy-ori3-1 cells were treated with *Bifidobacterium adolescentis* at gradient multiplicity of infection (MOI) values (0, 50, 100, 150, 200, 250) for 24 h, 48 h and 72 h, with the 0 MOI group set as the blank control group. After intervention, cells were digested with trypsin–EDTA solution (Genom, GNM25200), and the digestion reaction was terminated with complete medium. Cells were then collected by centrifugation for subsequent experiments.

#### 2.2.2 Cell function analysis

Cell proliferative activity was assessed using the MTT assay. The absorbance of each well was detected at 570 nm with an ELISA microplate reader (Awareness, USA). Cell cycle distribution of Nthy-ori3-1 cells was analyzed using a commercial Cell Cycle Staining Kit (MultiSciences Biotech, China). Total cellular proteins were extracted using RIPA lysis buffer supplemented with fresh protease and phosphatase inhibitor cocktails (Beyotime Biotechnology). The protein concentration of each sample was quantified using a BCA protein assay kit (Beyotime Biotechnology, Shanghai, China) to ensure consistent loading for subsequent protein detection.

### 2.3 Mouse model and experimental design

#### 2.3.1 Animal model and *Bifidobacterium* intervention

Five-week-old female BALB/c mice were purchased from Charles River Laboratories. All mice were housed in a specific pathogen-free (SPF) animal facility with a controlled environment (temperature: 22 ± 2 °C, humidity: 60 ± 5%, 12 h light/dark cycle). Mice were fed with standard chow diet and had free access to sterile drinking water. All animal experimental procedures were approved by the Animal Ethics Committee of Wenzhou Medical University (Approval No. wydw2023-2076).

Recombinant adenovirus encoding the 1–289 amino acid fragment of thyroid-stimulating hormone receptor (AD-TSHR289) and empty control adenovirus (AD-NULL) were obtained from Hanbio Biotechnology. After one week of adaptive feeding, mice were randomly divided into four groups (*N* = 8 per group): blank control group (CONTROL), empty adenovirus group (AD-NULL), GD model group (AD-TSHR289), and *Bifidobacterium* -intervened GD model group (AD+Bf).

For GD model establishment, mice in the AD-NULL group were injected with 1.0 × 10^8^ PFU empty adenovirus, while mice in the AD-TSHR289 and AD+Bf groups were injected with 1.0 × 10^8^ PFU AD-TSHR289 at week 0, week 4 and week 6. Mice in the control group received an equal volume of sterile PBS. From week 0 to week 6, mice in the AD+Bf group were intragastrically administered with *Bifidobacterium adolescentis* at a dose of 1.0 × 10^9^ CFU/mL (200 μL per mouse per day), while other groups received equal volumes of sterile PBS daily. The successful construction of GD hyperthyroidism model was verified by detecting serum T4 and TRAb levels.

#### 2.3.2 Thyroid hormone evaluation

Serum levels of thyroxine (T4) and thyroid-stimulating hormone receptor antibody (TRAb) were measured to confirm hyperthyroidism in AD-TSHR289 and AD+Bf groups. Corresponding commercial ELISA kits were used for the detection in accordance with the manufacturer’s instructions. All samples were tested in duplicate. The optical density (OD) values at 450 nm were recorded via a microplate reader, and the concentrations of target indicators were calculated based on the standard curve.

#### 2.3.3 Histopathological examination

The body weight of mice in each group was recorded every 3 days throughout the experimental period. At week 9, all mice were euthanized, and blood samples were collected and centrifuged to isolate serum. Thyroid tissues, intestinal tissues and intestinal feces were harvested subsequently. All tissue specimens were fixed in 10% neutral buffered formalin, embedded in paraffin, and sectioned continuously. Tissue sections were stained with hematoxylin and eosin (H&E; Sigma-Aldrich, USA) following standard protocols. Histopathological morphological changes of thyroid and intestinal tissues, as well as intestinal barrier integrity, were observed and photographed under a light microscope for subsequent pathological evaluation. Thyroid pathological changes, including follicular hyperplasia, follicular cavity dilation, and epithelial cell proliferation, as well as intestinal inflammatory infiltration, villus damage and intestinal barrier disruption, were observed and photographed under an optical microscope.

### 2.4 RT-qPCR

Total RNA was isolated from tissue samples via grinding homogenization, and 2 μg of qualified RNA was collected for subsequent reverse transcription. RNA was reverse transcribed into cDNA using a preconfigured reaction kit following standard protocols. qPCR amplification was performed with a three-step program: 30s initial denaturation at 95 °C, 40 cycles of 5 s denaturation at 95 °C plus 60s annealing/extension at 60 °C, and a final dissociation curve analysis. Primer sequences are detailed as below. Relative mRNA expression levels of inflammatory factors were calculated using the 2-ΔΔCt method. CD40-forwardprimer: AGAAGGCTGGCACTGTACGA. GAPDH-forwardprimer: AGGTCGGTGTGAACGGATTTG.

### 2.5 Fecal microbiota analysis via 16S rRNA gene sequencing

Fresh fecal samples were aseptically collected from hyperthyroidism patients before and after ^131^I treatment, then immediately frozen at −80 °C. Genomic DNA was extracted with QIAGEN DNA Extraction Kit following the manufacturer’s protocols. The 16S rRNA V3-V4 region was amplified, and its processing included quality control of amplified products, purification, and library construction before sequencing. Beta diversity was analyzed via UniFrac, and sequencing data were processed using QIIME 1.9.0. OTUs (97% sequence similarity) were annotated against the Greengenes database, with those below 0.005% total abundance excluded. LEfSe analysis was applied to evaluate intestinal flora variability. Linear discriminant analysis (LDA) effect size (LEfSe) analysis was conducted to identify microbial features with biological consistency and statistical significance.

### 2.6 Western blotting

Thyroid and colon tissues were homogenized in RIPA lysis buffer, followed by 10 min of ice incubation and centrifugation at 12,000 × g for 30 min at 4 °C. BCA Protein Assay Kit was used to determine protein concentrations, which were then adjusted appropriately. After separation by SDS-PAGE, proteins were transferred onto membranes, blocked with 5% skim milk for 2 h, and incubated with primary antibodies at 4 °C overnight. The primary antibodies employed were as follows: anti-ZO-1 (1:1000, Diagbio, China), anti-Occludin (1:1000, Abways, China), anti-IL-6 (1:1000, Diagbio, China), *β*-Actin (1:3000, Abways, China), GAPDH (1:2000, Abways, China), Cyclin B1 (1:2000, Abways, China), β-catenin (1:1000, Abways, China), C-Myc (1:1000, Diagbio, China), CDK1 (1:2000, Abways, China), TNF-*α* (1:500, Biodragon, China), P-65 (1:1000, Abways, China), NIS (1:1000, Diagbio, China), P-p65 (1:1000, Abways, China), IL-1β (1:1000, Diagbio, China), and MUC2 (1:1000, Diagbio, China). After washing the membranes, they were incubated with secondary antibodies (1:2000, Beyotime, China) for 2 h at room temperature. Protein expression was detected using an ultrasensitive ECL chemiluminescence kit and a chemiluminescence imaging system. ImageJ software was used to quantify the grayscale intensity of protein bands, which were normalized to the corresponding internal controls and statistically analyzed with GraphPad Prism 7.0 software.

### 2.7 ELISA

All reagents were equilibrated to room temperature prior to the assay, and serial dilutions of the standard samples were prepared. A volume of 50 μL of standards and test samples was added to the corresponding wells separately, followed by 50 μL of biotin-labeled antibody. The plate was sealed to avoid evaporation and incubated at 37 °C for 60 min. After incubation, the solution in each well was discarded, and the wells were washed three times with wash buffer. Subsequently, 80 μL of streptavidin-HRP conjugate was added to each well, and the plate was incubated at 37 °C for another 30 min. After removing the reagents and washing the wells repeatedly, chromogenic substrate was added, followed by incubation at 37 °C in the dark for 10 min. Finally, 50 μL of stop solution was added to each well, and the optical density (OD) value was measured at a wavelength of 450 nm.

### 2.8 Statistical analysis

Experiments should be repeated at least 3 times, and graphing and statistical analysis calculated using Graphpad Prism 7.0. Cell cycle detection and data analysis were performed using a CytExpert 2.3 flow cytometer. All Data were expressed as mean±standard deviation. Microbial analysis and physiological index analysis were performed using Kruskal Wallis test, while other statistics were analyzed using t-test. *p* values <0.05 represent significant differences.

## 3 Results

### 3.1 Participants

According to the diagnostic criteria for GD, a total of 17 GD patients were enrolled, including 13 females and 4 males, aged 16–60 years old. All patients done thyroid function blood test before ^131^I radiotherapy and showed increased level of T3, T4, TRAb. Individual ^131^I doses were given by experienced nuclear medicine doctors (dose= $\frac{\text{weight of thyroid gland}\times 100\mathrm{uCi}/g}{\max\%\text{uptake of thyroid}}$ ). All patients had seen the improvement of symptom and fall of T3, T4 level (Table 1).

**Table 1:** Clinical characteristics of the participants.

Characteristics	Pre ^131^I radiotherapy	Post ^131^I radiotherapy	*p* value
Age (y)	44.41 ± 11.4		
Thyroid weight (g)	40.31 ± 15.97		
FT4	37.50 ± 19.22	15.24 ± 10.21	0.000*
FT3	15.17 ± 8.69	6.44 ± 2.91	0.001*
TRAb	77.41 ± 150.16	66.68 ± 120.15	0.828

* *p* < 0.05.

### 3.2 Changes of the gut microbiome in GD patients

**Figure 1:**
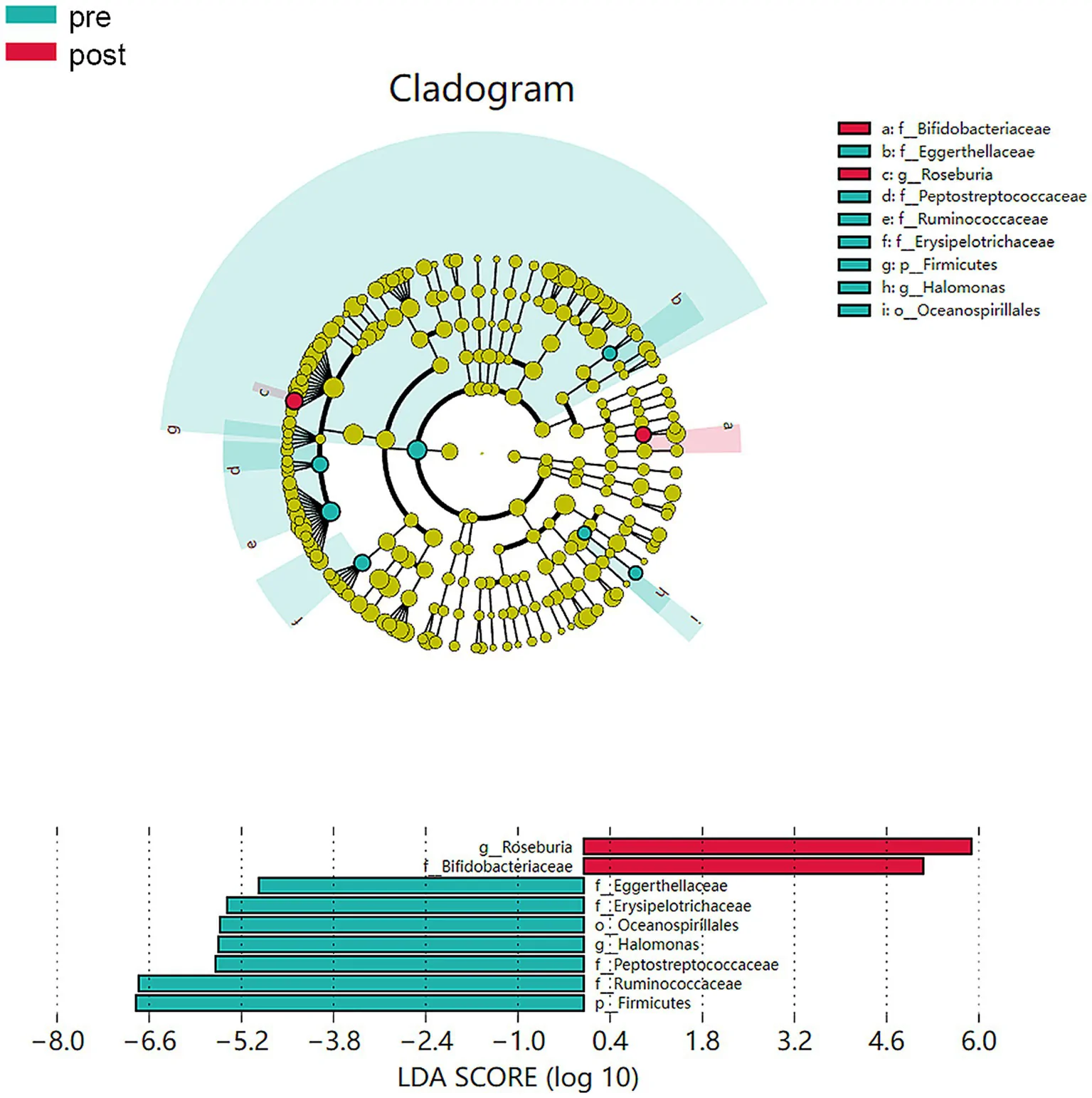
Changes of the gut microbiome in GD patients. Before ^131^I treatment, *Bifidobacteriaceae* and *Roseburia* were highly abundant in GD patients. After ^131^I treatment, *Eggerthellaceae*, *Peptostreptococcaceae*, *Ruminococcaceae*, *Erysipelotrichaceae*, *Firmicutes*, *Halomonas*, and *Oceanospirillales* were significantly enriched.

Using LEfSe to explore the phylogenetic spectrum of specific bacterial groups and major bacteria, we found that *Bifidobacteriaceae* and *Roseburia* were highly abundant in GD patients before ^131^I treatment, *Eggerthellaceae*, *Peptostreptococcaceae*, *Ruminococcaceae*, *Erysipelotrichaceae*, *Firmicutes*, *Halomonas*, and *Oceanospirillales* were significantly enriched after ^131^I treatment (Figure 1A). At the taxonomic level, *Bifidobacteriaceae* and *Roseburia* were notably abundant in GD patients before ^131^I treatment, while *Eggerthellaceae*, *Erysipelotrichaceae*, *Oceanospirillales*, *Halomonas*, *Peptostreptococcaceae*, *Ruminococcaceae*, and *Firmicutes* were significantly enriched after ^131^I treatment (LDA score > 2) (Figure 1B).

### 3.3 The effect of *Bifidobacterium* on the proliferation ability of thyroid cells

**Figure 2:**
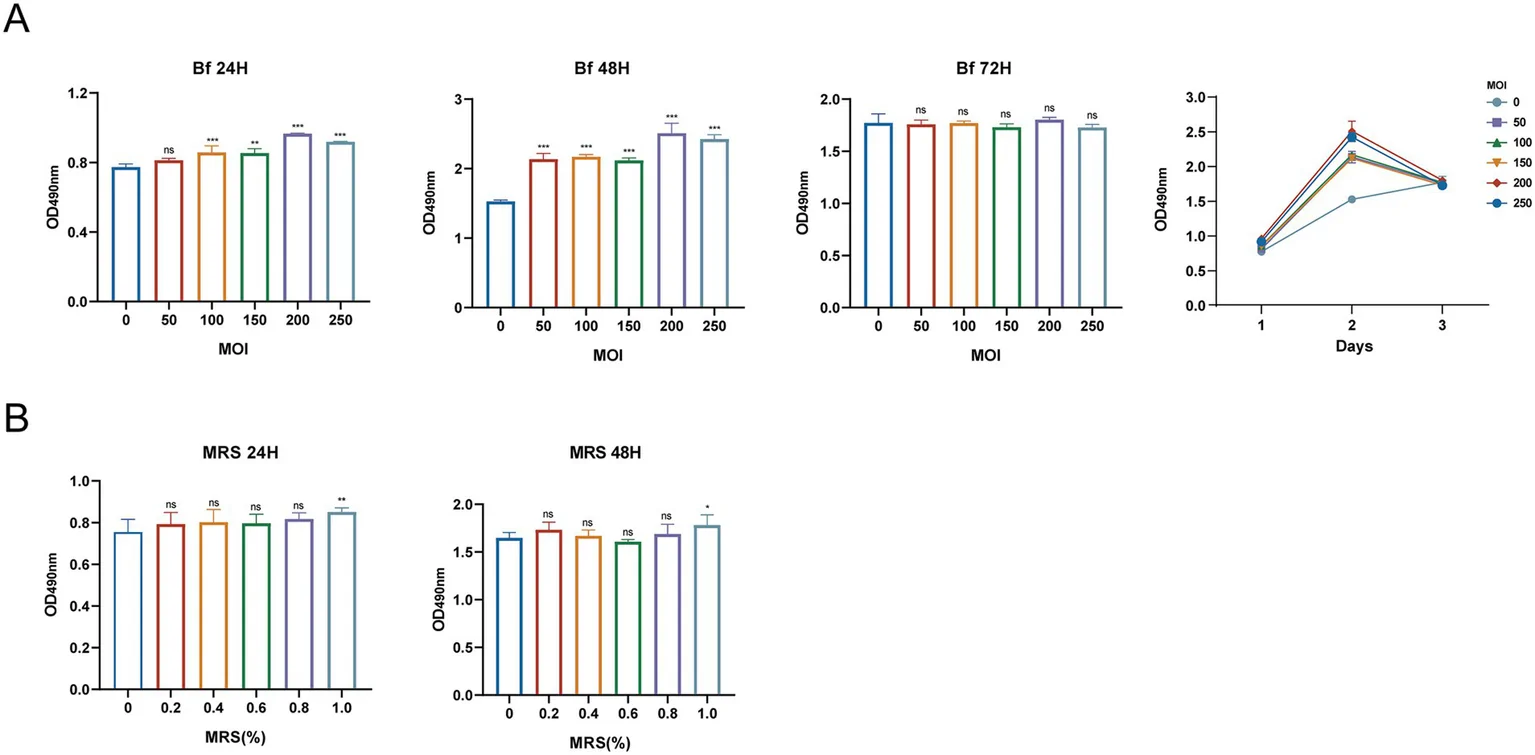
The effect of *Bifidobacterium* with different MOIs on the proliferation ability of thyroid cells. **(A)** After 24 and 48 h of co-treatment with *Bifidobacterium*, the proliferation of thyroid cells increased with the higher MOI. After 72 h of co-treatment with *Bifidobacterium*, no significant differences were observed in thyroid cells among different MOI groups. **(B)** After 24 and 48 h of co-treatment with MRS, there was no significant proliferation in thyroid cells between different concentrations. *p < 0.05; ***p < 0.001.

After 24 and 48 h of co-treatment with *Bifidobacterium*, the proliferation ability of thyroid cells increased with the higher MOI of *Bifidobacterium*. However, no significant differences in cell proliferation were observed at 72 h (Figure 2A). The most pronounced effect was noted with 200 MOI after 48 h co-treatment with *Bifidobacterium*. Additionally, the MTT assay using MRS medium as control group showed that MRS medium did not significantly promote thyroid cell proliferation (Figure 2B).

**Figure 3:**
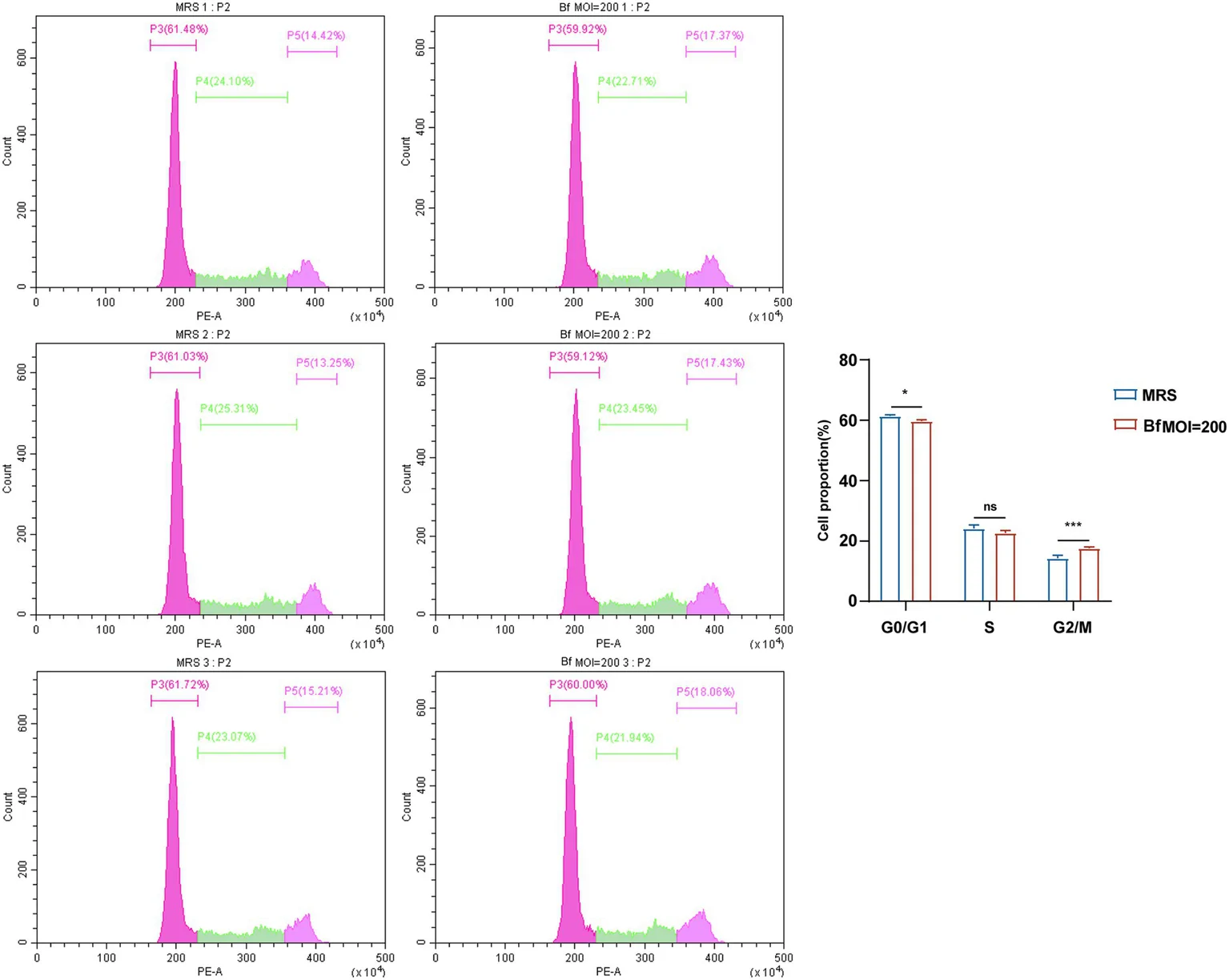
The effect of *Bifidobacterium* on the cell cycle of thyroid cells. Compared to the MRS medium control group, after 48 h co-treatment with *Bifidobacterium* (MOI = 200), the proportion of cells in the G0/G1 and S phases were decreased, the proportion of cells in the G2 phase increased. *p < 0.05; **p < 0.01; ***p < 0.001.

Moreover, co-treatment of thyroid cells with *Bifidobacterium* (MOI = 200) for 48 h resulted in a decrease in the proportion of cells in G0/G1 and S phases, while proportion of cells in G2 phase increased (Figure 3). These results imply that *Bifidobacterium* has a stimulatory effect on the cell cycle of thyroid cells, enhancing their progression through the cycle, which could contribute to increased cellular proliferation.

### 3.4 Measurement of related proteins in thyroid tissues after *Bifidobacterium* treatment

**Figure 4:**
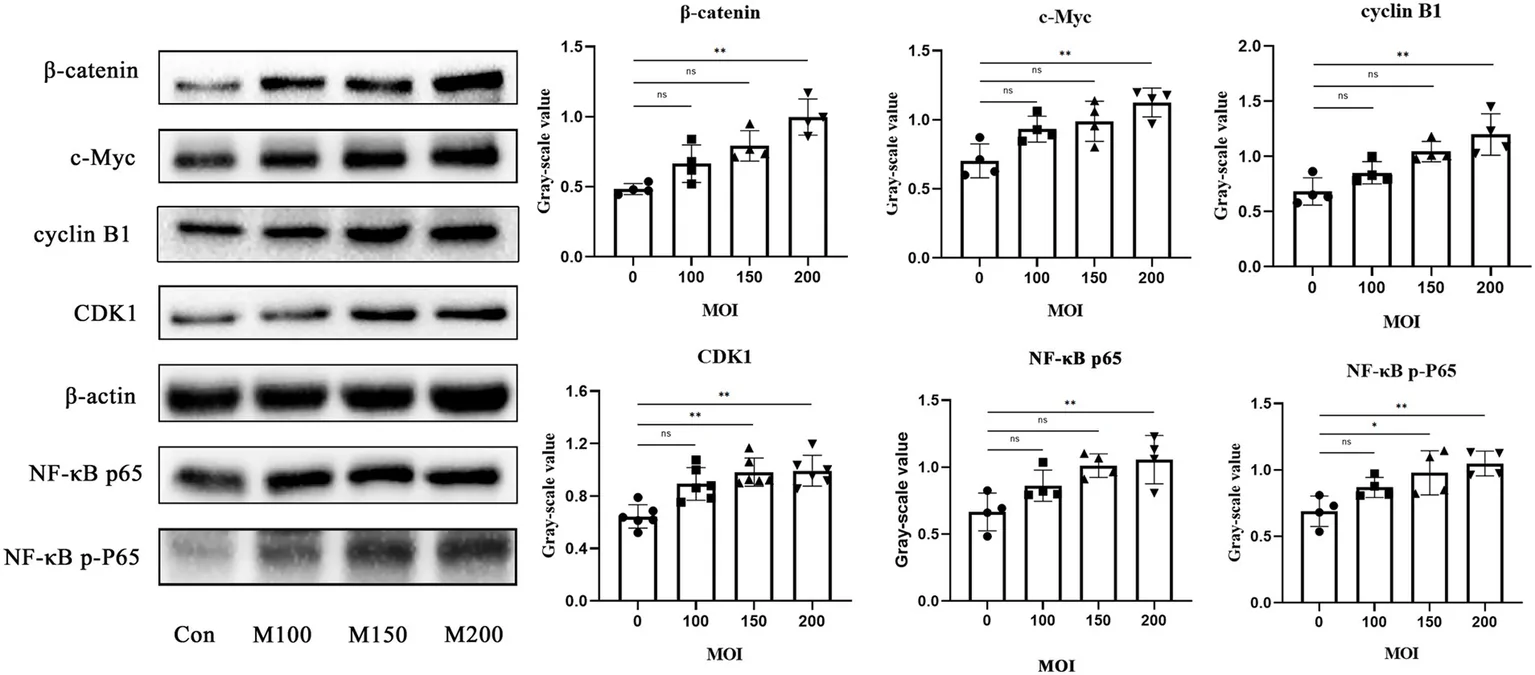
*Bifidobacterium* promotes the proliferation of thyroid cells and the expression of NF-κB pathway-related proteins. Western blotting was performed to detect the protein levels in the control group and groups treated with MOI 100 (M100), 150 (M150), and 200 (M200). *β*-actin was used as the internal loading control. Statistical analysis was performed with Dunnett’s multiple comparisons test to compare the treatment groups with the control group. At MOI = 200, β-catenin, cyclin B1, CDK1, and c-Myc increased with higher MOI values (*p* < 0.01). At MOI = 0/100/150/200, NF-κB subunits such as P-65 and p-P65 increased with higher MOI values (*p* < 0.01).

The expression levels of proliferation and cell cycle-related proteins, including *β*-catenin, cyclin B1, CDK1, and c-Myc, gradually increased with higher *Bifidobacterium* MOI values. Similarly, NF**-**κB subunits such as P-65 and p-P65, also exhibited a dose-dependent upregulation (Figure 4).

### 3.5 *Bifidobacterium* promotes disease progression in GD mouse

**Figure 5:**
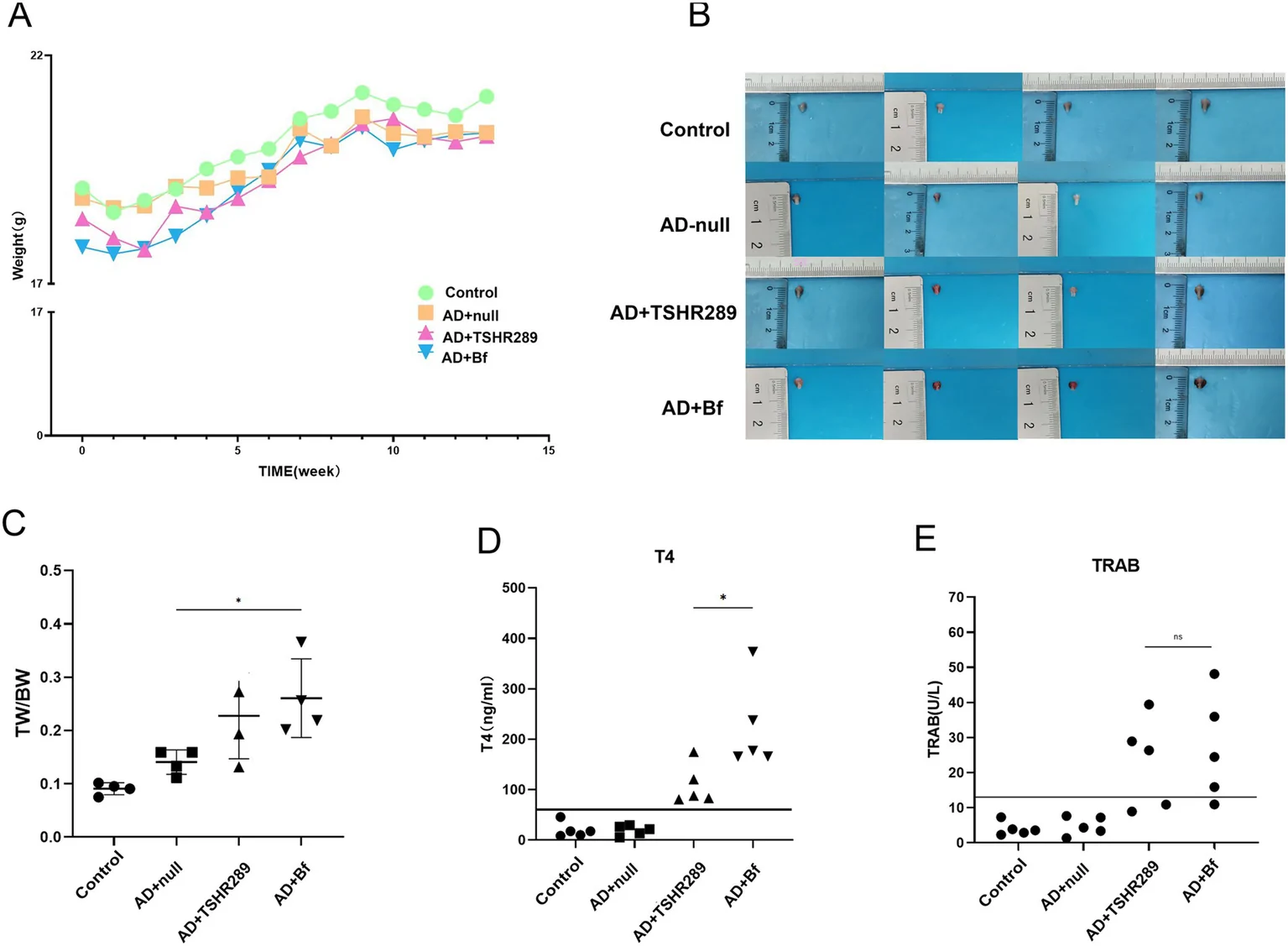
*Bifidobacterium* promotes the disease progression of GD mice. **(A)** Body weight of mice in the control group, AD-NULL group, AD-TSHR289 group, and AD+Bf group was measured every 3 days throughout the 13-week immunization period. The body weight of mice in all groups showed a steady increasing trend over time. **(B)** Representative gross morphology of thyroid glands isolated from mice in each group at the endpoint of the experiment. Compared with the control group, obvious thyroid enlargement was observed macroscopically in the other three experimental groups. **(C)** Quantification of thyroid weight-to-body weight (TW/BW) ratio, showing significantly elevated values in the AD+Bf groups compared to the AD-NULL groups (*p* < 0.05). **(D)** T4 levels were significantly increased in AD-TSHR289 and AD+Bf mice relative to controls (*p* < 0.05). **(E)** TRAb levels, the pathogenic autoantibody driving GD, were elevated in AD-TSHR289 and AD+Bf groups, with no statistically significant difference between groups Dunnett’s multiple comparisons test (*p* > 0.05).

Figure 5A shows the body weight change trend over 13 weeks of immunization. Representative thyroid images revealed significant morphological differences between the AD-TSHR289 group and AD+Bf group versus the control and AD-NULL groups (Figure 5B). A relative increase in thyroid weight/body weight (TW/BW) was observed in the AD-TSHR289 group and AD+Bf group, indicating thyroid enlargement, a hallmark of GD pathology (Figure 5C). ELISA results showed that mice in AD-TSHR289 group and AD+Bf group had increased level of T4 and TRAb (Figures 5D,E), indicating that mice in AD-TSHR289 group and AD+Bf group been hyperthyroidism status.

### 3.6 The impact of *Bifidobacterium* on thyroid tissue of GD mice

**Figure 6:**
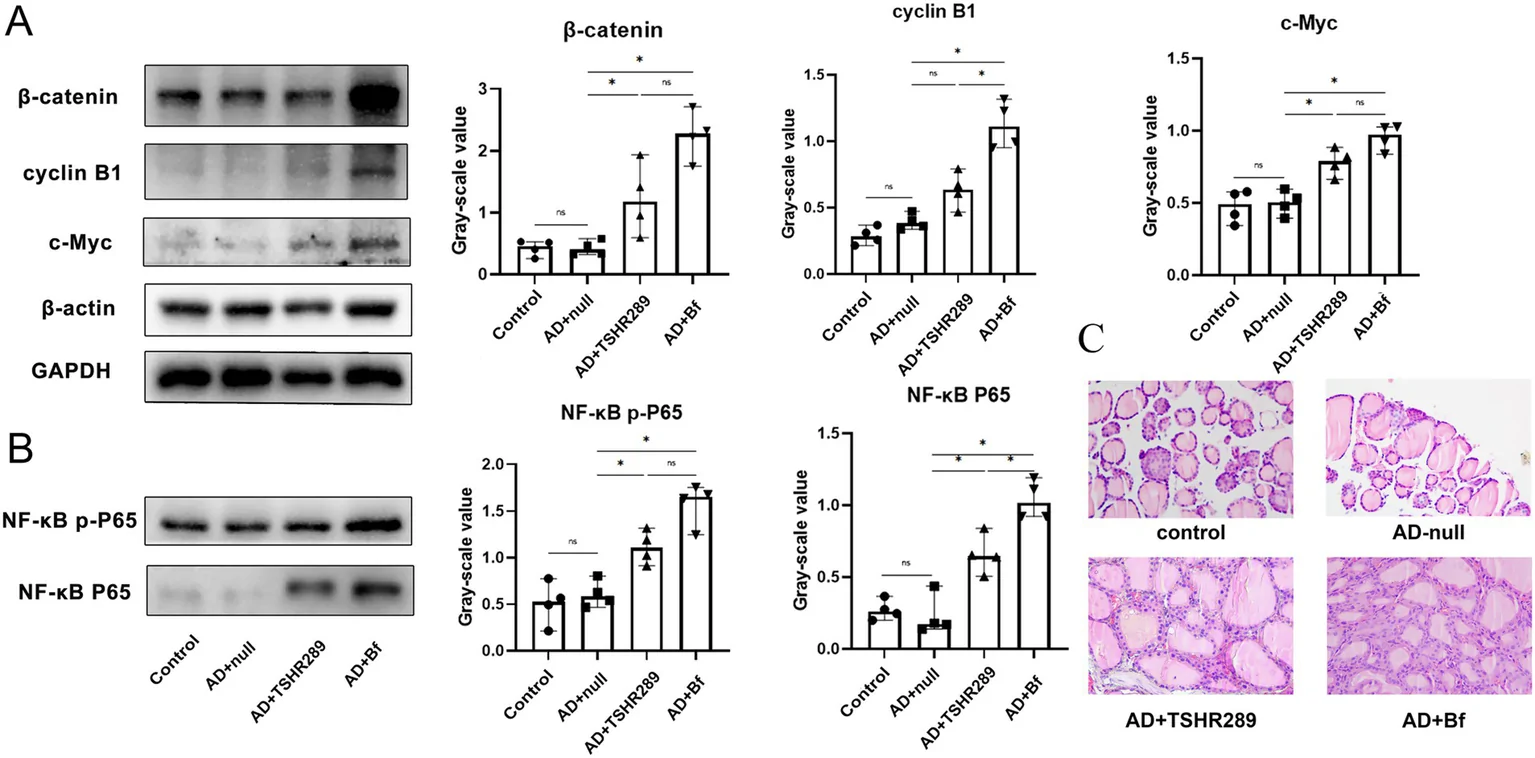
The impact of *Bifidobacterium* on thyroid tissue of GD mice. **(A)** Compared with the control group and AD-NULL group, β-catenin, c-Myc, cyclin B1, and were more expressed in AD-TSHR289 group and AD+Bf group (*p* < 0.05). **(B)** Compared with the control group and AD-NULL group, NF-κB P65 and p-P65 were more expressed in AD-TSHR289 and AD+Bf group (*p* < 0.05). **(C)** Thyroid follicles in the control and AD-NULL groups exhibited normal architecture, AD-TSHR289 group showed abnormal hyperplasia of thyroid follicular cells and chronic thyroid enlargement. The AD+Bf group displayed exacerbated thyroid follicular hyperplasia and tissue swelling compared to the AD-TSHR289 group. *p < 0.05.

Compared with the control group and AD-NULL group, cell proliferation and cycle related proteins, such as *β*-catenin, c-Myc, cyclin B1, and were more expressed in AD-TSHR289 group of thyroid tissues, and even more significant in AD+Bf group (Figure 6A). Proteins associated with the NF**-**κB pathway (P65 and p-P65) also exhibited a dose-dependent increase in expression, indicating pathway activation (Figure 6B). Thyroid follicles in the control and AD-NULL groups exhibited normal architecture without hyperplasia or chronic enlargement. But mice in the AD-TSHR289 group showed abnormal hyperplasia of thyroid follicular cells and chronic thyroid enlargement, consistent with GD pathology. The AD+Bf group displayed exacerbated thyroid follicular hyperplasia and tissue swelling compared to the AD-TSHR289 group (Figure 6C).

### 3.7 The impact of *Bifidobacterium* on colon tissue of GD mice

**Figure 7:**
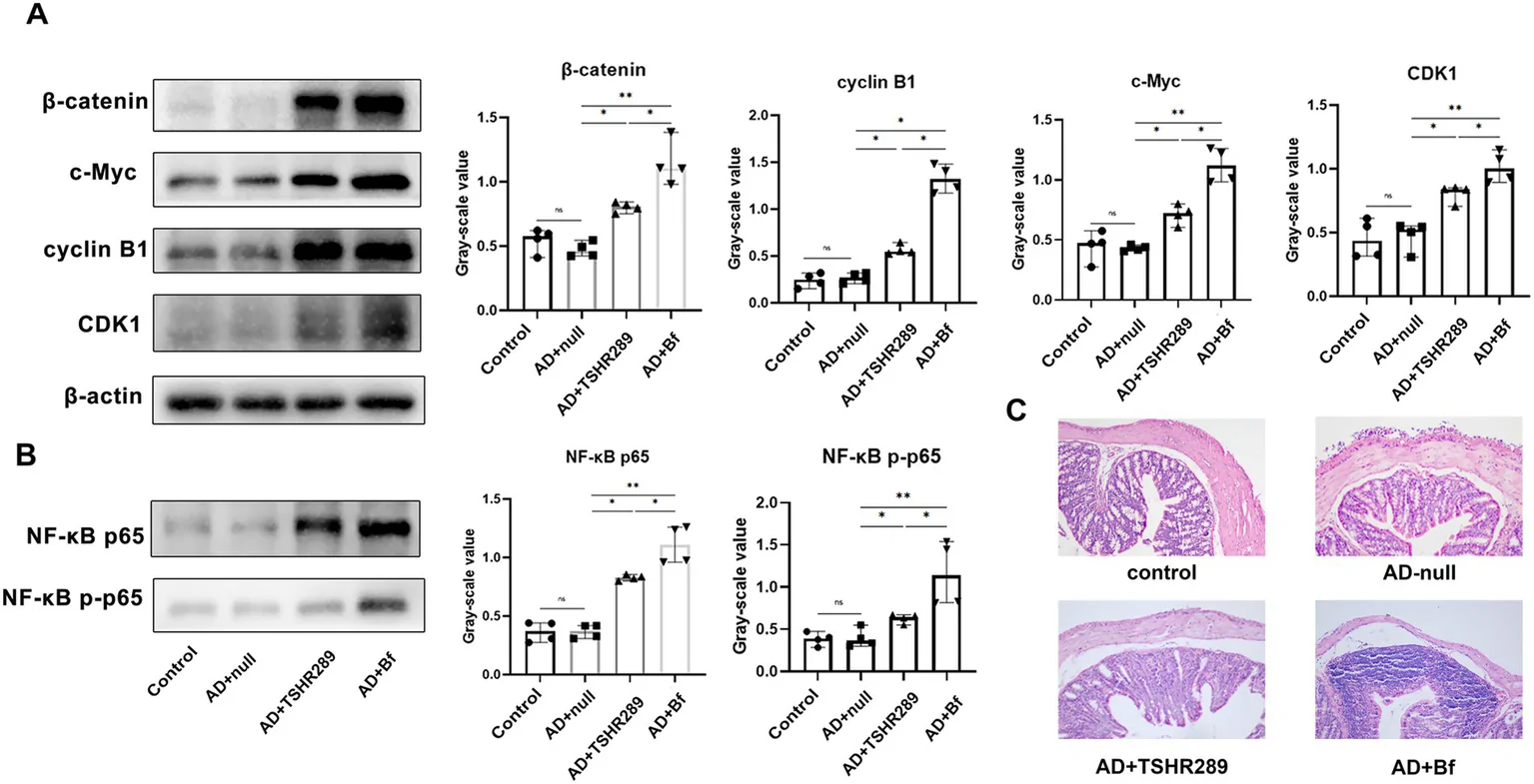
The impact of *Bifidobacterium* on colon tissue of GD mice. **(A)** Compared with the control group and AD-NULL group, β-catenin, cyclin B1, CDK1, and c-Myc were more expressed in AD-TSHR289 group and AD+Bf group (*p* < 0.05). **(B)** Compared with the control group and AD-NULL group, NF-κB P65 and p-P65 were more expressed in AD-TSHR289 and AD+Bf group (*p* < 0.05). **(C)** The AD-TSHR289 and AD+Bf group exhibited crypt architectural distortion, mucosal damage, and moderate inflammatory cell infiltration on colon tissues. *p < 0.05; **p < 0.01.

Cell proliferation and cycle related proteins, such as *β*-catenin, cyclin B1, CDK1, and c-Myc, were more expressed in AD-TSHR289 group of colon tissues, and even more in AD+Bf group (Figure 7A). NF**-**κB pathway related proteins such as P65 and p-P65, also exhibited a dose-dependent increase in expression, indicating pathway activation (Figure 7B). Colon tissues in these groups retained intact crypt architecture, undamaged intestinal mucosa, and minimal inflammatory cell infiltration. The AD-TSHR289 group exhibited crypt architectural distortion, mucosal damage, and moderate inflammatory cell infiltration, suggesting GD-associated gut pathology. Colon tissues in the AD+Bf group showed more severe alternations compared to the AD-TSHR289 group (Figure 7C).

**Figure 8:**
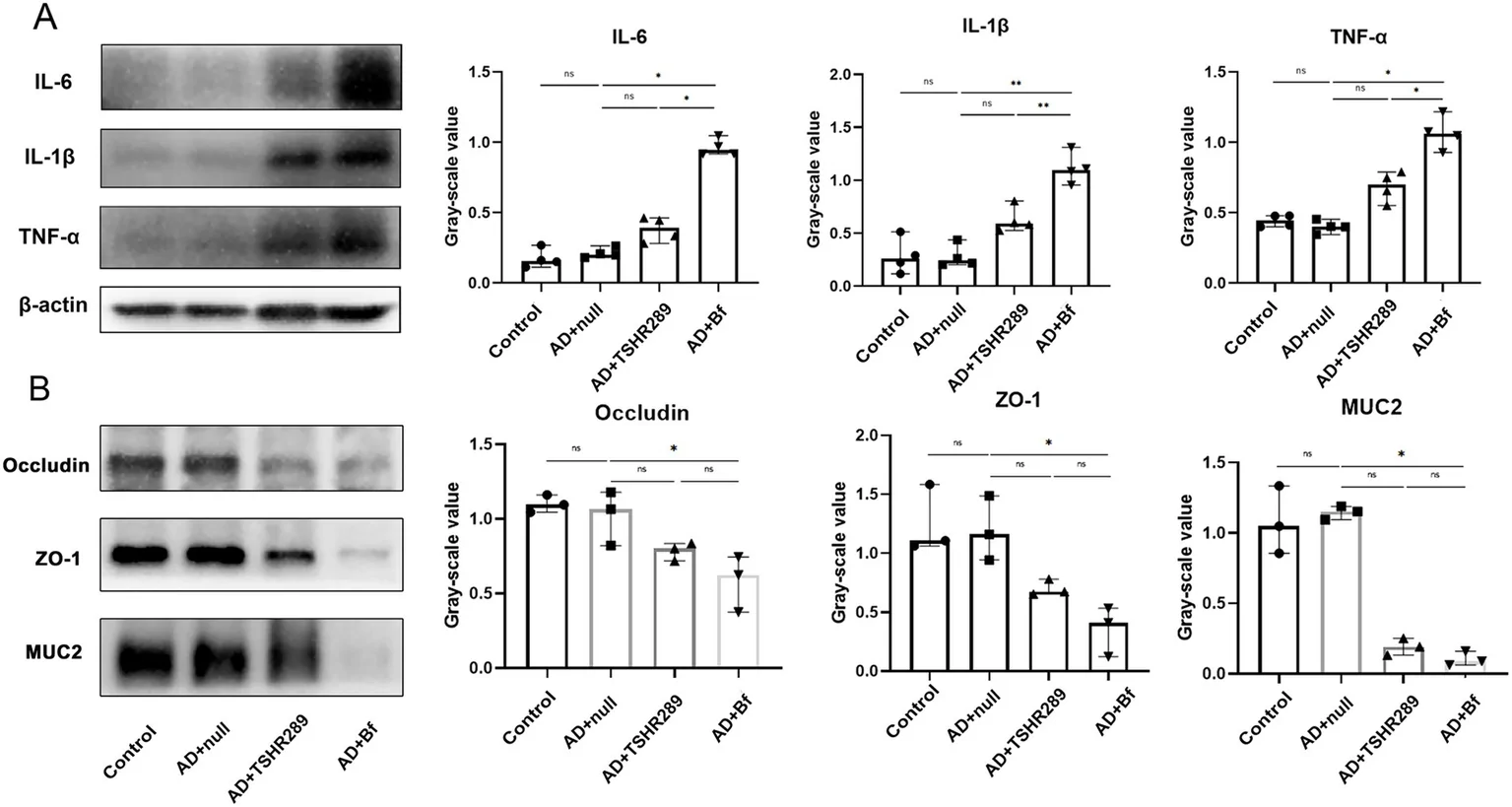
The impact of *Bifidobacterium* on expression of inflammatory factor in colon tissue of GD mice. **(A)** Compared with the control group and AD-NULL group, IL-6 (*p* < 0.05), IL-1β (*p* < 0.01), and TNF-*α* (*p* < 0.05) significantly increase in AD-TSHR289 and AD+Bf group. **(B)** Compared with the control group and AD-NULL group, Occludin, ZO-1, MUC2 were significantly increased in AD-TSHR289 group and AD+Bf group (*p* < 0.05). *p < 0.05; ** *p* < 0.01.

The expression levels of inflammatory factors IL-6, IL-1β, and TNF-*α* significantly increase in both AD-TSHR289 group and AD+Bf group, with AD+Bf group being higher (Figure 8A). We found that expressions of Occludin, ZO-1, MUC2 were significantly increased in samples of colon tissue with AD-TSHR289 group and AD+Bf group when compared with control and AD-NULL group. At the same time, the expression levels of intestinal barrier protein related proteins in the model group and the bacterial treatment group decreased (Figure 8B).

### 3.8 The impact of *Bifidobacterium* on expression of CD40 and NIS

**Figure 9:**
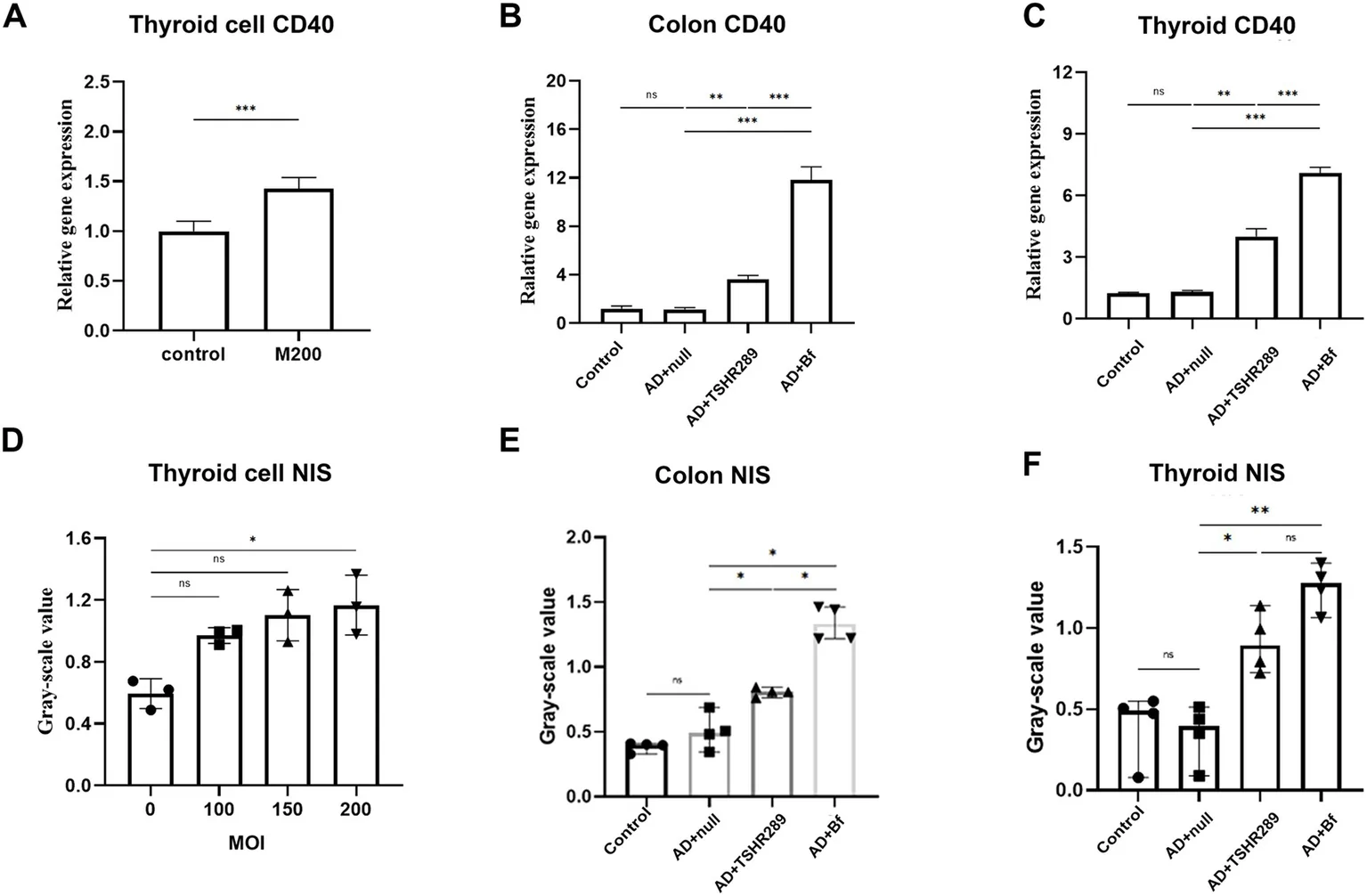
The impact of *Bifidobacterium* on expression of CD40 and NIS. **(A)** At MOI of 200, CD40 (*p* < 0.001) expression in thyroid cell were significantly increase compared with control group. **(B,C)** Compared with the control group and AD-NULL group, CD40 expression in thyroid tissues and colon tissues were significantly increase in AD-TSHR289 and AD+Bf group (*p* < 0.05). **(D)** At MOI = 0/100/150/200, NIS expression in thyroid cell increased with higher MOI values (*p* < 0.05). **(E,F)** Compared with the control group and AD-NULL group, NIS expression in thyroid tissues and colon tissues were significantly increase in AD-TSHR289 and AD+Bf group (*p* < 0.05). *p < 0.05; **p < 0.01; ***p < 0.001.

Notably, CD40 and NISexpression in thyroid cell, thyroid tissues and colon tissues were confirmed rising both in AD-TSHR289 group and AD+Bf group (Figure 9).

### 3.9 The impact of *Bifidobacterium* on the composition of gut microbiota in GD mice

**Figure 10:**
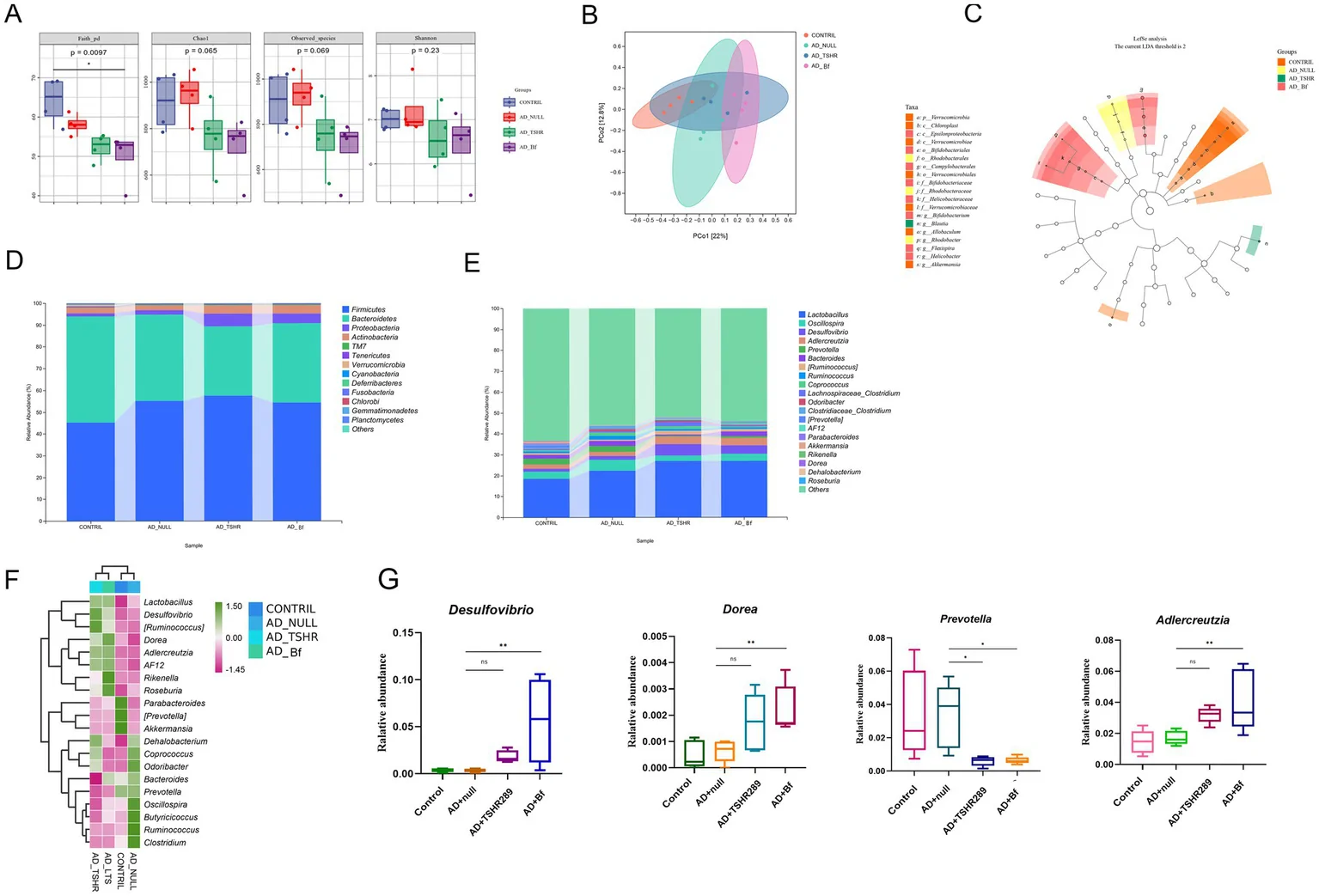
The impact of *Bifidobacterium* on the composition of gut microbiota in GD mice. **(A)** Alpha diversity analysis: Faith_pd, Chao1, observed_species, and Simpson index. **(B)** Beta diversity analysis: PCoA reveals distinct clustering of gut microbiota structure among the four groups of mice. **(C)** LEfSe analysis: Significantly different microbial taxa among the four groups are displayed. **(D)** Relative abundance bar plot of mouse gut microbiota at the phylum level, showing the top 20 most abundant phyla among the four groups. **(E)** Relative abundance bar plot of mouse gut microbiota at the genus level, showing the top 20 most abundant genera among the four groups. **(F)** Heatmap of species composition at the genus level across different groups. **(G)** Relative abundances of *Desulfovibrio*, *Dorea*, *Prevotella*, and *Adlercreutzia* in each group (*p* < 0.05). *p < 0.05; **p < 0.01.

Alpha diversity analysis revealed that although there were no significant differences in species richness (Observed Species) and evenness (Shannon index) between control group and GD group, the GD group showed a significant decrease in Faith PD (*p* < 0.05), indicating impaired phylogenetic diversity of its microbial community (Figure 10A). Beta diversity analysis revealed significant differences in microbial community structure between control group and GD group. In the PCoA plot, the four groups of samples were clearly separated, indicating that disease status significantly influenced gut microbiota composition (Figure 10B). Further LEfSe analysis identified a significant increase in the abundance of *Blautia* in AD-TSHR289 group, while *Epsilonproteobacteria*, *Bifidobacterials*, *Campylobacterales*, *Bifidobacteraceae*, *Helicobacteraceae*, *Bifidobacterium*, *Flexispira* and *Helicobacter* were enriched in AD+Bf group (Figure 10C). Relative abundance bar plot of mouse gut microbiota at the phylum level and genus level showed that separation in gut microbial community structure among the four mouse groups (Figures 10D–H).

## 4 Discussion

The gut is increasingly recognized as a significant endocrine organ, playing an often overlooked but crucial role in the onset and progression of GD. We characterized the microbiome changes in GD patients before and after radioactive treatment, and explored the influence of *Bifidobacterium adolescentis* on thyroid cells and GD mice. Our data demonstrate that *Bifidobacterium adolescentis* promotes the growth of thyroid cells and exacerbates hyperthyroidism through the activation of the CD40-NF-κB-NIS signaling pathway.

Recent studies have shown that GD patients exhibit reduced diversity and abundance of certain microbiota (Song et al., 2024; Ishaq et al., 2018; Chao et al., 2025). We found similar alterations in the gut microbiome of GD patients who underwent ^131^I radiotherapy. Surprisingly, our data revealed a significant decline in *Bifidobacterium* abundance as the concentrations of T3 and T4 recovered to healthy levels. While some studies reported decreased *Bifidobacterium* levels in GD patients (Song et al., 2024; Ishaq et al., 2018; Chao et al., 2025; Liu et al., 2023), others found elevated *Bifidobacterium* abundances in GD patients, which were positively correlated with the severity of disease activity (Liu et al., 2024; Jiang W. et al., 2021; Ji et al., 2022; Song et al., 2019). Moreover, GD patients treated with methimazole exhibited a reduction in *Bifidobacterium* abundance with improved thyroid function (Huo et al., 2021). Furthermore, a study on patients who underwent thyroidectomy for thyroid cancer, followed by postoperative radioiodine therapy and subsequent hypothyroidism, revealed a significant reduction in *Bifidobacterium* (Zhou et al., 2024). Although ionizing radiation is known to damage the intestinal epithelial barrier and mucus layer, it does not appear to negatively affect the human gut microbiota or influence the abundance of *Bifidobacterium* (Tong et al., 2022; Li et al., 2023). Therefore, we propose that changes in *Bifidobacterium* abundance are correlate with thyroid function.

Previous research has shown that fecal microbiota transplanted from GD patients significantly increases the incidence of GD in mice, elevating serum thyroid hormone and proinflammatory cytokine levels (Su et al., 2020). This highlights the critical role of gut microbiota imbalances in the pathogenesis of GD, suggesting that these alterations are not merely secondary effects or coincidental phenomena. Changes in *Bifidobacterium* abundance in patients with newly diagnosed GD have been positively correlated with TRAb, TGAb, and TPOAb (Wagner et al., 2023). We found the coordinated upregulation of cell cycle regulators and NF-κB pathway components, implying that *Bifidobacterium adolescentis* enhances thyroid cell proliferation and activates proliferative signaling pathways. The role of the microbiota in autoimmune thyroid diseases is thought to involve cross-reactivity between certain microbial antigens and self-antigens (Liu et al., 2023; Ji et al., 2022; Kiseleva et al., 2011). *Bifidobacterium* shares amino acid sequences with thyroid peroxidase and thyroglobulin. This mimicry may contribute to triggering GD through a molecular simulation mechanism.

In our experiment, we observed an increase of CD40 expression in both thyroid and colon tissues of GD mice. The association between CD40 and GD has been confirmed in multiple studies across different ethnic and geographic populations (Gong et al., 2021; Samimi and Haghpanah, 2020). Increased CD40 expression on thyroid cells has been linked to the autoimmune response targeting the thyroid in GD (Huber et al., 2012; Lee et al., 2023). Furthermore, several studies have suggested that CD40 polymorphisms can predict the remission and relapse of GD (Wang et al., 2013; Inoue et al., 2012). Transgenic mouse models that constitutively overexpress thyroidal CD40 develop more severe experimental autoimmune GD and thyrotoxicosis. In contrast, blocking CD40 stimulation in these animal models suppresses the progression to overt thyroiditis (Carayanniotis et al., 1997; Ye et al., 2012). Similarly, functional blockade of CD40 using a murine antibody effectively prevents clinical expression in an animal model of multiple sclerosis (Boon et al., 2001). Additionally, we found that NF-κB expression was elevated in both thyroid and intestinal tissues, suggesting that activation of the NF-κB signaling pathway may play a significant role in the pathogenesis of GD. As previously reported in thyroid cells, CD40 signaling can activate NF-κB in orbit fibrocytes in Graves’ orbitopathy (Boon et al., 2020). NF-κB is a protein complex crucial for regulating cell proliferation and survival (Cai et al., 2014). We believe that activating CD40-NF-κB pathways may promote thyroid cell proliferation.

The thyroid and gastrointestinal epithelium both originate from the endoderm and share functional and morphological similarities, which allow them to regulate each other and maintain homeostasis in both systems (Yan et al., 2024). Short-chain fatty acids produced by *Bifidobacterium* can inhibit histone deacetylase, activating NIS expression and enhancing iodine uptake (Fernandes et al., 2023; Lin et al., 2022; Zheng et al., 2022). Our findings demonstrate that *Bifidobacterium adolescentis* significantly upregulates the expression of NIS in thyroid cells, which could potentially enhance the uptake of iodine and alleviate the hyperthyroid state associated with GD. The gut microbiota and its metabolites, often referred as the thyroid-gut axis, may influence the permeability and integrity of the gut barrier, thereby affecting thyroid hormones metabolism (Desai et al., 2016). Our study found evidence of crypt structure damage, intestinal mucosal injury, and inflammatory cell infiltration in the colon tissue of GD mice. Disruptions in gut microbiota balance can lead to intestinal epithelial barrier dysfunction, allowing antigens to pass into the circulation and activate the immune system (Sun et al., 2024; Cayres et al., 2021). In our study, the expression levels of inflammatory factors were significantly elevated in both the GD model and bacterial treatment groups, with the bacterial treatment group showing higher levels. Pivotal inflammatory mediators such as IL-17, TNF-*α*, and IL-6 are integral to the pathophysiology of GD (Liu et al., 2024).

While the study provides valuable insights into the potential role of *Bifidobacterium* in GD, there are some limitations to consider. The study involved a relatively small number of GD patients, and larger sample sizes are needed to confirm the findings and establish a more robust correlation between *Bifidobacterium* levels and GD. While the GD mouse model used in the study is a valuable tool, it may not fully recapitulate the complexity of human GD. Additional studies in humans are needed to confirm the findings and assess the clinical relevance of the observed effects. Another limitation of the current study is that only NIS protein expression was detected, whereas functional iodide uptake assays were not performed.

## 5 Conclusion

In conclusion, these findings suggest that *Bifidobacterium* may contribute to the pathogenesis of GD by activating the CD40-NF-κB signaling pathway and modulating NIS expression. The gut microbiota appears to play a significant role in GD, and targeting specific bacterial species like *Bifidobacterium* could potentially offer novel therapeutic strategies for managing this condition.

## Data Availability

The datasets generated and analyzed during the current study are available in the NCBI repository under BioProject accession PRJNA1371336.
